# A Dream

**Published:** 1892-09

**Authors:** 


I had a funny dream the other night. I was standing at the corner of a magnificent new building in this city, admiring the architecture, when I saw a gentlemen coming up the street with a large bottle in his hand, with the magic letters K. R. G. in large type marked on one side. In a moment, another smiling, complacent-looking gentlemen, whom I had noticed coming out of the building with a glass jar under his arm, the contents of which I could not recognize, met the former in front of me, and not until they spoke, did I recognize them. As they were so close, I could not help but overhear their conversation, which was as follows:

DR. MEDICINE: "Good evening, Dr. Surgery."

DR. SURGERY: "How are you, Dr. Medicine? What's new in your line?"

DR. MEDICINE: "New! Why you must not read the papers. Don't you know we have reached the millenium in medicine? See that bottle?"

DR. SURGERY: "I see three large letters on it."

DR. MEDICINE: "Ah! my boy, those letters stand for the name of this wonderful medicine by which the millenium has been reached. It has just been endorsed by a whole college. You don't believe it? Well, I will read you a letter from the president.

"'We have used King's Royal Germeteur in the Southern Female University during the past year. Among the many boarders, all expressed themselves as highly pleased, and I must say that not an adverse criticism was given. It was the almost universal remedy for every complaint among the young ladies.'

"How is that? What has Surgery got to say for herself?"

DR. SURGERY: "More than you, my boy. You should read the papers and keep up with the surgical progress. You see the contents of that jar?"

DR. MEDICINE: "Yes, what is it?"

DR. SURGERY: "That is an appendix—a vermiform appendix! Cut it out of a man the other day. The most wonderful and delicate operation of the age; second one ever done in America. Read the account of it in the papers, and then you won't come talking to me about your millenium in medicine. If you did not see *the paper* I will get after the printer, for I told him to send out an extra edition."

DR. MEDICINE (as he poked Dr. Surgery in the ribs): "I see you are up to snuff; nothing like being in with the papers." And as they passed on with a mutual chuckle, I awoke from this horrid nightmare. W. F. W.

